# Hepatic CYP3A4 Enzyme Compensatively Maintains Endogenous Geranylgeranoic Acid Levels in *MAOB*-Knockout Human Hepatoma Cells

**DOI:** 10.3390/metabo12020140

**Published:** 2022-02-03

**Authors:** Yuki Tabata, Yoshihiro Shidoji

**Affiliations:** 1 Department of Nutrition, Kiryu University, Midori 379-2392, Gunma, Japan; tabata-yu@kiryu-u.ac.jp; 2 Molecular and Cellular Biology, Graduate School of Human Health Science, University of Nagasaki, Nagayo 851-2195, Nagasaki, Japan

**Keywords:** CYP3A4, geranylgeraniol, geranylgeranoic acid, hepatoma, cancer prevention, isoprenoids, retinoids, diterpenoid

## Abstract

Geranylgeranoic acid (GGA), developed as a preventive agent against second primary hepatoma, has been reported to be biosynthesized via the mevalonate pathway in human hepatoma-derived cells. Recently, we found that monoamine oxidase B (MAOB) catalyzed the oxidation of geranylgeraniol (GGOH) to produce geranylgeranial (GGal), a direct precursor of endogenous GGA in hepatoma cells, using tranylcypromine, an inhibitor of MAOs, and knockdown by *MAOB* siRNA. However, endogenous GGA level was unexpectedly unchanged in *MAOB*-knockout (KO) cells established using the CRISPR-Cas9 system, suggesting that some other latent metabolic pathways maintain endogenous GGA levels in the *MAOB*-KO cells. Here, we investigated the putative latent enzymes that oxidize GGOH in Hep3B/*MAOB*-KO cells. First, the broad-specific cytochrome P450 enzyme inhibitors decreased the amount of endogenous GGA in Hep3B/*MAOB*-KO cells in a dose-dependent manner. Second, among the eight members of *cytochrome P450* superfamily that have been suggested to be involved in the oxidation of isoprenols and/or retinol in previous studies, only the *CYP3A4* gene significantly upregulated its cellular mRNA level in Hep3B/*MAOB*-KO cells. Third, a commercially available recombinant human CYP3A4 enzyme was able to oxidize GGOH to GGal, and fourth, the knockdown of *CYP3A4* by siRNA significantly reduced the amount of endogenous GGA in Hep3B/*MAOB*-KO cells. These results indicate that CYP3A4 can act as an alternative oxidase for GGOH when hepatic MAOB is deleted in the human hepatoma-derived cell line Hep3B, and that endogenous GGA levels are maintained by a multitude of enzymes.

## 1. Introduction

Geranylgeranoic acid (all-*trans* 3,7,11,15-tetramethyl-2,6,10,14-hexadecatetraenoic acid or GGA) has been developed as a preventive agent against the second primary hepatoma [1,2]. In terms of the molecular and cellular mechanisms for its preventive action, we have previously reported that GGA induces cell death in human hepatoma-derived cells via several cellular processes, including (i) endoplasmic reticulum stress response [3], (ii) incomplete autophagic response [4], and (iii) Toll-like receptor 4 (TLR4)-mediated pyroptosis [5].

Previously, we also reported that GGA is a natural compound found in some medicinal herbs, such as turmeric [6], and later showed that GGA is an endogenous lipid present in various organs of male Wistar rats, not only in plants and but also in animal tissues [7]. Historically, however, GGA was first reported by Fliesler et al. as a major metabolic labeled product when bovine retinal homogenate [8] and its tissue culture [9] were incubated with radioisotope-labeled mevalonate. In addition, a similar report was made about a parasite of *Schistosoma mansoni* [10], and later, a whole genome analysis confirmed that the sterol biosynthetic system is defective in this animal [11]. Recently, we observed, by isotopomer spectral analysis, that GGA is biosynthesized from mevalonate via farnesyl diphosphate and geranylgeranyl diphosphate (GGPP) in human hepatoma-derived cells [7]. 

In studies of enzymes involved in GGA biosynthesis, we very recently reported that monoamine oxidase B (MAOB) plays an important role in the biosynthesis of GGA using HuH-7 and Hep3B cells derived from human hepatoma [12]. MAOB is involved in the oxidation of geranylgeraniol (GGOH), which provides the formation of geranylgeranial (GGal), a direct precursor of GGA synthesis. GGPP, an intermediate in polyisoprenoid biosynthesis, has previously been shown to be converted to GGOH by GGPPase in rat liver homogenates [13]. Inhibition of MAOB activity by the treatment with tranylcypromine (TCP), as a MAO inhibitor, or knockdown of the *MAOB* gene expression by siRNA treatment significantly decreased the amount of endogenous GGA in human hepatoma-derived cell lines [12], strongly suggesting that hepatic MAOB is an essential enzyme for endogenous GGA biosynthesis.

However, when the *MAOB* gene was knocked out by the CRISPR-Cas9 system in Hep3B cells (Hep3B/*MAOB*-KO cells), the amount of endogenous GGA was not reduced, contrary to our expectation [12]. However, back-transfection of the MAOB expression plasmid into the Hep3B/*MAOB*-KO cells restored the downregulation of the endogenous GGA levels by *MAOB* siRNA. These results indicate that when MAOB is normally expressed in human hepatocytes, the endogenous level of GGA is dependent on MAOB activity. Therefore, in Hep3B/*MAOB*-KO cells, other enzymes may be involved in maintaining the amount of endogenous GGA in a compensatory manner in place of the defective MAOB, but the other enzymes acting in place of MAOB are still unknown. Our previous study using HuH-7 cells showed that GGOH oxidation activity is present in the microsomal fraction as well as in the mitochondrial fraction where MAOB is localized, but is not detected in the cytosolic fraction [14]. In fact, knockdown experiments using cytosolic alcohol dehydrogenase 1A (*ADH1A*, reported as GGOH dehydrogenase [15])-specific siRNA confirmed that the amount of GGA in Hep3B/*MAOB*-KO cells was not reduced [12]. On the other hand, a group of cytochrome P450 enzymes localized to the hepatic microsomal fraction are known to be able to oxidize isoprenols such as geraniol, farnesol, GGOH, and retinol [15,16,17]; however, whether cytochrome P450 enzymes are involved in the oxidation of GGOH in *MAOB* knockout hepatoma cells has not been investigated.

Here, we investigated the involvement of cytochrome P450 enzymes, a representative hepatic microsomal enzyme, as an alternative to MAOB in Hep3B/*MAOB*-KO cells. Analysis of several cytochrome P450 superfamily members revealed that CYP3A4, a major component of hepatic cytochrome P450 enzymes, is involved in the oxidation of GGOH in Hep3B/*MAOB*-KO cells.

## 2. Results

### 2.1. Downregulation of Endogenous GGA in Hep3B/MAOB-KO Cells by Cytochrome P450 Enzyme-Inhibitors

**Figure 1:**
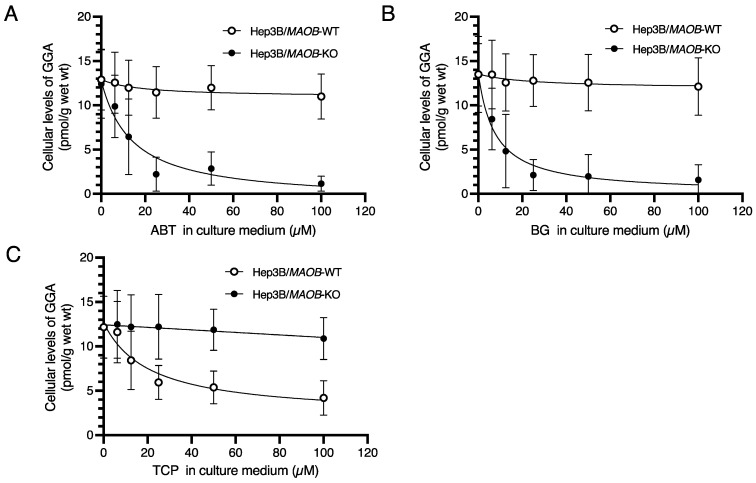
Cytochrome P450 enzyme inhibitors-induced downregulation of cellular GGA levels in Hep3B/*MAOB*-KO cells. Dose-dependent changes of endogenous GGA level in Hep3B/*MAOB*-KO cells (closed circle) and Hep3B/*MAOB*-WT cells (open circle) after treatment with 0–100 μM of ABT (**A**), BG (**B**) or TCP(**C**) for 24 h. The amount of the intracellular GGA represents the mean ± SD in triplicate. The IC_50_ of ABT, BG or TCP was obtained using GraphPad Prism 9.3. GGA, geranylgeranoic acid; MAOB, monoamine oxidase B; ABT, 1-aminobenzotriazole; BG, bergamottin; TCP, tranylcypromine.

First, we examined the sensitivity of endogenous GGA levels in Hep3B/*MAOB*-KO cells to cytochrome P450 enzyme inhibitors. A well-known nonspecific and irreversible inhibitor of cytochrome P450 enzymes [18], 1-aminobenzotriazole (ABT), dose-dependently decreased the endogenous GGA content in Hep3B/*MAOB*-KO cells with an IC_50_ (the half maximal inhibitory concentration) of 10.9 µM, whereas it did not affect the endogenous GGA levels in *MAOB* wild-type Hep3B cells (Hep3B/*MAOB*-WT cells) (Figure 1A). Bergamottin (BG), another broad-specific inhibitor of cytochrome P450 enzymes, also dose-dependently reduced the amount of endogenous GGA only in Hep3B/*MAOB*-KO cells with an IC_50_ of 7.5 µM (Figure 1B), but not in Hep3B/*MAOB*-WT cells. Conversely, TCP, an inhibitor of MAOB and some cytochrome P450 enzymes such as CYP2A6 [19], dose-dependently reduced the amount of endogenous GGA only in Hep3B/*MAOB*-WT cells (IC_50_ of 21.0 µM), but did not decrease the level of endogenous GGA in Hep3B/*MAOB*-KO cells at all (Figure 1C).

### 2.2. Expression Levels of the CYP3A4 Gene Were Specifically Upregulated in Hep3B/MAOB-KO Cells

**Figure 2:**
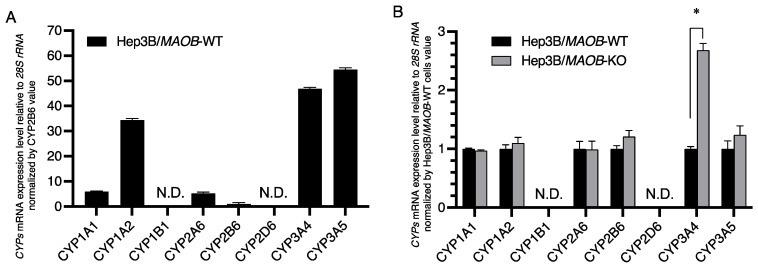
The relative expression levels of each cytochrome P450 enzyme involved in lipid metabolism and/or retinoid metabolism in Hep3B/*MAOB*-WT and Hep3B/*MAOB*-KO cells. (**A**) The cellular mRNA levels of each *cytochrome P450* gene are depicted relative to the *CYP2B6* mRNA level in Hep3B/*MAOB*-WT cells. (**B**) The cellular mRNA levels of each *cytochrome P450* gene in Hep3B/*MAOB*-KO cells are normalized by the corresponding respective *cytochrome P450* mRNA level in Hep3B/*MAOB*-WT cells. Each bar represents the mean ± SEM (*n* = 3). * *p* < 0.05 compared with control (Hep3B/*MAOB*-WT). N.D.: Not detected.

Next, we measured the cellular mRNA levels of some *cytochrome P450* superfamily members, which are targets of BG and have been reported to be involved in oxidation of isoprenols [16] and/or retinol [17]. In Hep3B/*MAOB*-WT cells, the *CYP3A5* mRNA level was the highest among the *cytochrome P450* superfamily members measured, followed by *CYP3A4* and *CYP1A2*. Then, *CYP1A1* and *CYP2A6* were about one-tenth of *CYP3A5*, *CYP2B6* was one-fiftieth of *CYP3A5*, and *CYP1B1* and *CYP2D6* were not detected at all (Figure 2A). Compared with Hep3B/*MAOB*-WT cells, Hep3B/*MAOB*-KO cells significantly increased only the levels of *CYP3A4* mRNA but maintained the cellular each mRNA level of other *cytochrome P450* superfamily members tested such as the *CYP1A1, 1A2, 2A6, 2B6* and even *3A5* genes (Figure 2B).

### 2.3. Catalytic Activity of Recombinant Human CYP3A4 in the Oxidation of GGOH to GGal

**Figure 3:**
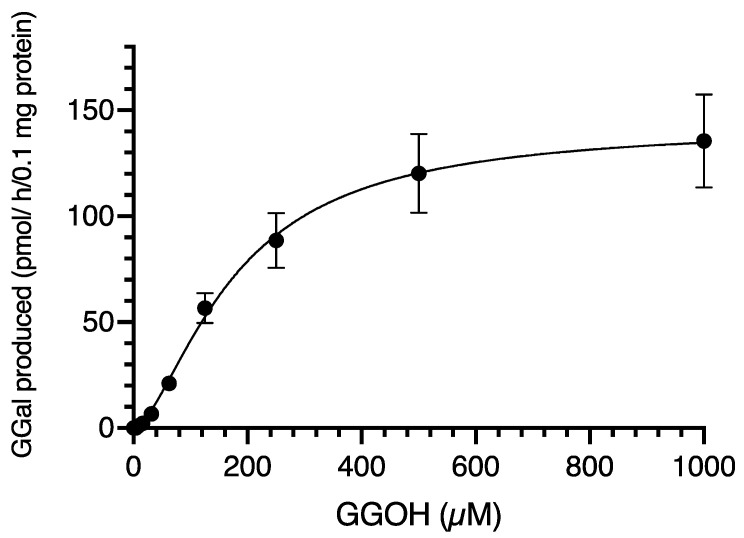
Recombinant human CYP3A4 catalyzed oxidation of GGOH to GGal. The increasing concentrations of GGOH were incubated with recombinant hCYP3A4 (0.1 mg protein) with 5 mM NADPH at 37 °C for 1 h. The amounts of GGal products were measured by LC/MS/MS analysis. Each point represents the mean ± SD (*n* = 3). The titration curve was drawn by allosteric sigmoidal enzyme kinetics in GraphPad Prism 9.3 software. GGal, geranylgeranial; GGOH; geranylgeraniol.

Because the results obtained so far in this study strongly indicate that the CYP3A4 enzyme is involved in the oxidation of GGOH to GGal in Hep3B/*MAOB*-KO cells, we next decided to examine whether recombinant human CYP3A4 protein (rhCYP3A4) can catalyze the oxidation reaction of GGOH. We used a commercially available CYP3A4 catalytic system with active rhCYP3A4, co-expressed with active NADPH (the reduced form of nicotinamide adenine dinucleotide phosphate) reductase for enzyme assay. The rhCYP3A4 enzyme oxidized GGOH to GGal, giving non-Michaelis–Menten-type kinetics when incubated at 0–1000 µM GGOH (Figure 3). The titration curve of the enzyme activity with GGOH apparently shows a noticeable sigmoid. Therefore, allosteric sigmoidal enzyme kinetics were selected in GraphPad Prism 9.3 software and the titration curve was approximated using the equation of $Y=V_{max}\times X^{h}÷\left(K_{prime}+X^{h}\right)$ (r^2^ = 0.9297), where the kinetics parameters were *h* = 1.598 and *K*_prime_ = 3888 µM. The *h* coefficient is greater than 1, indicating that the curve is sigmoidal due to positive cooperativity.

### 2.4. Downregulation of Endogenous GGA by CYP3A4 siRNA Transfection

Since several lines of evidence were obtained that CYP3A4 oxidizes GGOH to GGal, we finally conducted knockdown experiments using *CYP3A4*-specific siRNA in a cell culture system to analyze whether the knockdown of the *CYP3A4* gene reduces the amount of endogenous GGA. 

**Figure 4:**
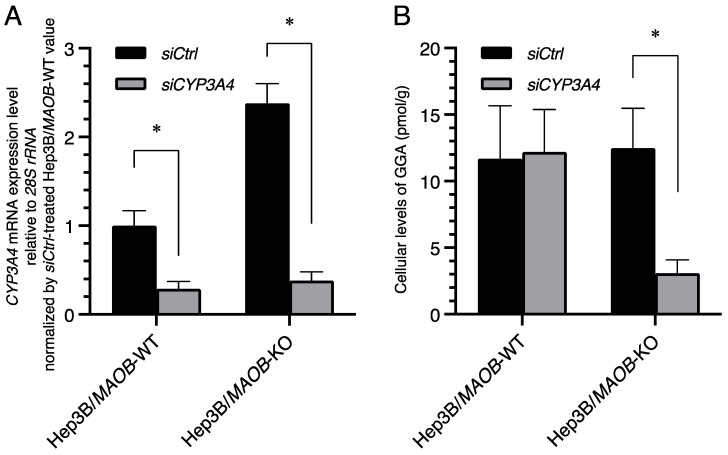
*CYP3A4* siRNA-induced downregulation of cellular GGA levels in Hep3B/*MAOB*-KO cells. (**A**) The relative expression levels of *CYP3A4* mRNA to that of *siCtrl*-treated Hep3B/*MAOB*-WT cells upon *MAOB* siRNA treatment in Hep3B/*MAOB*-WT or Hep3B/*MAOB*-KO for 72 h. Each bar represents the mean ± SEM (*n* = 3). (**B**) The endogenous GGA levels of the lipid extract from Hep3B/*MAOB*-WT or Hep3B/*MAOB*-KO after incubation with *CYP3A4* siRNA for 120 h. The amount of the intracellular GGA represents the mean ± SD of three measurements. * *p* < 0.05 compared with *siCtrl*. *siCtrl*, non-targeting scrambled siRNA (negative control); *siCYP3A4*, siRNA specific for *CYP3A4* mRNA; GGA, geranylgeranoic acid.

The *CYP3A4* mRNA levels in Hep3B/*MAOB*-KO and Hep3B/*MAOB*-WT cells transfected with *CYP3A4* siRNA were significantly decreased 72 h after transfection (Figure 4A), but *CYP3A5*, a member of the *CYP3A* subfamily, was not knocked down at all by *CYP3A4* siRNA (Supplementary Figure S1). Following the decrease in the cellular *CYP3A4* mRNA levels, a significant decrease in endogenous GGA was found at 120 h after transfection in Hep3B/*MAOB*-KO cells (Figure 4B; *siCtrl* 12.48 ± 2.99 pmol/g; *siCYP3A4* 3.08 ± 1.01 pmol/g), but not in Hep3B/*MAOB*-WT cells.

## 3. Discussion

In the present study, we demonstrated that CYP3A4 oxidizes GGOH to GGal instead of MAOB in Hep3B/*MAOB*-KO cells, and that it is involved in the maintenance of endogenous GGA levels. The inhibition of cytochrome P450 enzyme activity by broad-specific inhibitors of cytochrome P450 enzymes significantly reduced endogenous GGA levels in Hep3B/MAOB-KO cells. The mRNA expression levels of *CYP3A4* in Hep3B/*MAOB*-KO cells were significantly upregulated compared to that of Hep3B/*MAOB*-WT cells, and knockdown of *CYP3A4* by siRNA significantly decreased the amount of endogenous GGA only in Hep3B/*MAOB*-KO cells. Furthermore, rhCYP3A4 oxidized GGOH to produce GGal, supporting the idea that CYP3A4 is involved in the synthesis of endogenous GGA.

The primary objective of this study was to find an enzyme that oxidizes GGOH instead of MAOB in Hep3B/*MAOB*-KO cells. Previous studies reported that several enzymes other than MAOB, such as ADH [15] and prenylcysteine oxidase 1 (PCYOX1) [20,21], can produce GGal, a direct precursor of GGA. However, a previous study that established Hep3B/*MAOB*-KO cells did not show the involvement of these two enzymes in the maintenance of endogenous GGA levels [12]. We previously investigated the GGOH oxidation activity of each subcellular fraction in human hepatoma-derived HuH-7 cells, and reported that in addition to the mitochondrial fraction where MAOB is localized, GGOH oxidation activity was also present in the microsomal fraction [14]. In the present study, we focused on cytochrome P450 enzymes, which are major enzymes localized in liver microsomes and whose GGOH oxidation activity has not yet been investigated.

First, we tested whether treatment with inhibitors of the cytochrome P450 enzymes in cell culture systems could reduce endogenous GGA levels. Both of the two non-selective cytochrome P450 enzyme inhibitors, ABT and BG, reduced the GGA level in Hep3B/*MAOB*-KO cells in a concentration-dependent manner, but neither inhibitor had much effect on endogenous GGA levels in Hep3B/*MAOB*-WT cells (Figure 1A,B). The two inhibitors used here both cause mechanism-based inhibition and are irreversible inhibitors, in which their respective metabolites covalently bind to cytochrome P450 enzymes [22,23]. Of note, BG and GGOH share a geranyl moiety in their molecular structures, suggesting that GGOH may compete with BG for the substrate binding site of cytochrome P450 enzymes. In other words, GGOH could be a substrate for cytochrome P450 enzymes. These findings suggest that cytochrome P450 enzymes are potential enzymes to oxidize GGOH when the *MAOB* gene is deleted. Treatment of Hep3B/*MAOB*-KO cells with TCP did not inhibit GGA production (Figure 1C). TCP is well known as a MAO inhibitor, but it is also known to act as an inhibitor against cytochrome P450 enzymes such as CYP1 and CYP2 subfamilies. However, since the IC_50_ of TCP for CYP3A4 is reported to be more than 166.7 µM and TCP has no apparent inhibitory effect on CYP3A4 [24], the result that GGA production could not be inhibited under the experimental conditions (≤100 µM TCP) of this study suggests that CYP3A4 among cytochrome P450 enzymes may be involved in GGA production in Hep3B/*MAOB*-KO cells.

Next, we selected *cytochrome P450* superfamily members that are inhibited by BG and that have been reported to oxidize isoprenols such as geraniol and retinol in previous studies [16,17,25,26], and measured their mRNA expression levels in Hep3B/*MAOB*-KO cells. Among the eight members measured here, only *CYP3A4* mRNA was significantly increased in Hep3B/*MAOB*-KO cells (Figure 2). The IC_50_ for CYP3A4 enzyme activity of ABT and BG has been reported to be approximately 10 µM [27] and 4.5 µM [28], respectively. On the other hand, the IC_50_ of the suppressive effect on endogenous GGA levels analyzed in the present paper is 10.9 µM for ABT and 7.5 µM for BG, respectively, which can be regarded as an inhibition of CYP3A4 enzyme activity. These findings strongly suggest that CYP3A4 is involved in the biosynthesis of endogenous GGA in Hep3B/*MAOB*-KO cells.

So, thirdly, we used rhCYP3A4 to investigate whether CYP3A4 actually oxidizes GGOH. The results showed that rhCYP3A4 does indeed oxidize GGOH to GGal, and moreover, the titration curve with GGOH shows allosteric sigmoidal-type kinetics (Figure 3). The Hill coefficient (*h*) was greater than 1, suggesting that rhCYP3A4 recognizes GGOH as a substrate in a dimeric or multimeric form that shows positive cooperativity. This is consistent with reports that CYP3A4 exerts catalytic activity in dimeric or multimeric form [29,30]. The *K*_prime_ of rhCYP3A4 for GGOH is approximately 4 mM, which is much higher than the *K*_m_ for GGOH of MAOB (34 μM) [12], indicating that GGOH is selected as a substrate for CYP3A4 only in the absence of MAOB. 

Finally, the knockdown experiment provided additional strong evidence that the *CYP3A4* gene is primarily responsible for the maintenance of cellular GGA levels in Hep3B/*MAOB*-KO cells. *CYP3A4* siRNA-mediated downregulation of the endogenous GGA levels caused more than 70% reduction in only Hep3B/*MAOB*-KO cells (Figure 4), suggesting that the majority of cellular GGA in Hep3B/*MAOB*-KO cells is produced through a *CYP3A4*-mediated process. In addition, to selectively detect the existence of CYP3A4-independent pathway of GGA production in Hep3B/*MAOB*-KO cells, we double-knocked down *CYP3A4* and *ADH1α* or *PCYOX1* with siRNA, but the endogenous GGA levels did not decrease any further compared to single-knockdown of *CYP3A4* in Hep3B/*MAOB*-KO cells (Supplementary Figure S2). In summary, although MAOB is primarily involved in the oxidation of GGOH in the generation and maintenance of endogenous GGA levels, the *CYP3A4* gene acts in place of *MAOB* to maintain endogenous GGA levels when the *MAOB* gene is defective.

Hepatic endogenous GGA levels are maintained primarily by the MAOB enzyme [12], and when the *MAOB* gene is deleted, the CYP3A4 enzyme is induced and acts to maintain endogenous GGA levels instead of the MAOB enzyme. The way that *MAOB*-gene expression relates to *CYP3A4* gene expression is currently unknown. Furthermore, the most curious question is why two enzyme systems, MAOB and CYP3A4, exist to maintain endogenous GGA levels. Hep3B cells, a cell line derived from human hepatocellular carcinoma, have been found to have almost the same concentration of endogenous GGA, 11.7 pmol/g in wild-type cells and 12.5 pmol/g in *MAOB* knockout cells. We need to clarify in the future why Hep3B cells need to maintain a GGA concentration of 12 pmol/g.

Finally, we would like to discuss the relationship between CYP3A4 enzyme and hepatoma from the viewpoint of inhibiting hepatocarcinogenesis, which is one of the biological activities of GGA. The liver normally expresses high levels of MAOB and biosynthesizes carcinogenic inhibitory GGA [7,12,31]. However, the enzymatic activity of MAOB is decreased at the site of hepatoma, and a classical paper described that the higher the grade of hepatoma, the lower the activity of MAOB [32]. Furthermore, when the prognosis of patients with hepatocellular carcinoma is compared between the high and low *MAOB* gene expression groups, the survival rate of the high *MAOB* gene expression group is higher (Supplementary Figure S3A) [33]. On the other hand, the *CYP3A4* gene is also highly expressed in the liver and is involved in maintaining the concentration of GGA when the *MAOB* gene is deficient, thus it is expected to have an inhibitory effect on hepatoma. In fact, a decrease in CYP3A4 expression at the microsomal fraction of clinical hepatomas has been reported [34] and when the prognosis of hepatoma patients was compared between the high and low expression groups of the *CYP3A4* gene, the survival rate was found to be higher in the high expression group, as in the case of *MAOB* (see Supplementary Figure S3B). Therefore, MAOB and CYP3A4, which are alternatively involved in the biosynthesis of hepatic GGA, are both hepatoma suppressive genes. There is one historical report that considers that benzylamine oxidase (equivalent to MAOB) may be involved in the degradation of carcinogenic amines of dietary origin to prevent hepatoma [32], but to the best of our knowledge, no metabolites other than GGA produced by enzymatic reactions of CYP3A4 or MAOB have ever been reported to inhibit the development of hepatoma.

In conclusion, here, we clearly demonstrate that hepatic CYP3A4 plays a new metabolic role in the maintenance of the bioactive isoprenoid lipid GGA levels in the hepatocytes under limited conditions of reduced MAOB activity.

## 4. Materials and Methods

### 4.1. Chemicals

GGA was prepared by Kuraray Co. (Okayama, Japan) and Kowa Pharmaceutical (Tokyo, Japan). GGOH was provided by Eisai Foods (99% pure; Tokyo, Japan). Acetonitrile (LC/MS grade), ethanol, dimethyl sulfoxide (DMSO), ABT, BG and TCP were purchased from Sigma-Aldrich (St. Louis, MO, USA). Methanol was purchased from Wako Pure Chemical Industries (Osaka, Japan). Chloroform was obtained from Kanto Chemical Co. (Tokyo, Japan). rhCYP3A4 was purchased from BioVision (Milpitas, CA, USA). All chemicals other than those stated above were of reagent grade.

### 4.2. Cell Culture

The Hep3B cell line (Hep3B/*MAOB*-WT cells) was obtained from DS Pharma Biomedical (Osaka, Japan). The Hep3B/*MAOB*-KO cell line was established in our previous study [12]. Hep3B/*MAOB*-KO and Hep3B/*MAOB*-WT cells were maintained in DMEM containing 5% FBS at 37 °C in a humidified atmosphere of 5% CO_2_.

### 4.3. Treatment of Cells with Cytochrome P450 Enzymes Inhibitors

Each cell (5 × 10^5^ cells/dish, in a 10 cm diameter dish) was inoculated and cultured in DMEM containing 5% FBS for 24 h; thereafter, the medium was replaced with FBS-free DMEM 1 day before ABT, BG or TCP treatment. After 24 h treatment of the cells with different concentrations (0–100 μM) of ABT, BG or TCP in DMSO cells were harvested using plastic cell lifter and cellular GGA was quantitatively measured using LC/MS/MS as described below. The IC_50_ of ABT, BG or TCP on the endogenous GGA levels was calculated using GraphPad Prism version 9.3.

### 4.4. RT-qPCR

Total RNA was prepared from each cell culture using the Fastgene™ RNA Basic kit (Nippon Genetics, Tokyo, Japan). For cDNA synthesis, Fastgene™ Scriptase II (Nippon Genetics) was used according to the manufacturer’s instructions. Real-time PCR was performed using LightCycler FastStart DNA MasterPLUS SYBR Green I (Roche Diagnostics, Tokyo, Japan) on a LightCycler 96 (Roche). Gene expression levels were analyzed using the 2^−ΔΔCt^ method. Primer sequences and real-time PCR settings used in this study are presented in Supplementary Tables S1–S3.

### 4.5. Chemical Synthesis and Purification of GGal

GGal was prepared and quantified as previously reported [12], and the aldehyde was used as a standard compound for LC/MS/MS.

### 4.6. Enzyme Assays

Aliquots of 0–1000 μM GGOH were incubated at 37 °C with the rhCYP3A4 protein (equivalent 0.1 mg protein each) and NADPH 5 mM in a final volume of 100 μL of phosphate buffered saline. The reaction was then stopped by chilling on ice, and the reaction mixture was diluted with 9 vol of ethanol. The resultant ethanolic extract was filtered through a Cosmonice Filter S cartridge (0.45 µm) prior to the analyses of GGal by LC/MS/MS. 

### 4.7. Transfection with siRNA

Readymade siRNA for the *CYP3A4* gene was purchased from SantaCruz Biotechnology (Santa Cruz, CA, USA). The siRNAs included three target specific 19–25 *nt* siRNAs designed to knock down the expression of a specific gene (see Supplementary Table S4). Transfection with siRNAs were performed in the procedure that we have previously reported.

### 4.8. Lipid Extraction and Quantitative Measurement of Cellular GGA and LC/MS/MS Analysis

Using the procedures described in our previous studies [7,12], total lipids extracted included GGA and GGal from each cell or reaction mixture and were measured by LC/MS/MS.

### 4.9. Statistical Analysis

The Shapiro–Wilk test was used to assess normality. Statistical comparisons were performed using ANOVA with a post hoc Scheffe test where appropriate. All data are presented with a statistically significant difference defined as *p* < 0.05. The SPSS statistical software package (Ver.25, IBM, Tokyo, Japan) was used for analysis.

## Data Availability

The data presented in this study are available on request from the corresponding author. The data are not publicly available due to ethical reason.

## Supplementary Materials

The following supporting information can be downloaded at: https://www.mdpi.com/article/10.3390/metabo12020140/s1, Figure S1: The expression levels of *CYP3A5* mRNA in Hep3B/*MAOB*-WT cells and Hep3B/*MAOB*-KO cells treated with *CYP3A4* siRNA for 72 h are shown as relative expression level to *CYP3A5* mRNA in *siCtrl*-treated Hep3B/*MAOB*-WT cells, Figure S2: Cellular GGA levels in Hep3B/MAOB-KO cells treated with *CYP3A4* siRNA and *ADH1α* siRNA or *PCYOX1* siRNA, Figure S3: Prognosis of hepatoma patients between the high and low expression groups of the *MAOB* or *CYP3A4* gene shown by Kaplan-Meier plots, Table S1: The nucleotide sequences of each primer used for real-time RT-qPCR, Table S2: The condition of thermal cycler for real-time RT-PCR, Table S3: The condition of thermal cycler for real-time RT-PCR of *28S rRNA*, Table S4: The sequences of each siRNA used for knockdown experiments.
