# Supplementary material for: Hepatic CYP3A4 Enzyme Compensatively Maintains Endogenous Geranylgeranoic Acid Levels in MAOB-Knockout Human Hepatoma Cells
Source: Metabolites. 2022 Feb 3;12(2):140. doi: 10.3390/metabo12020140 (PMC8880059; doi:10.3390/metabo12020140)
Supplement: Supplementary file 1 [file metabolites-12-00140-s001.zip › metabolites-1569307-supplementary.pdf]

Hepatic CYP3A4 enzyme compensatively maintains endogenous geranylgeranoic acid levels in *MAOB*-knockout human hepatoma cells.

Yuki Tabata[1,2], Yoshihiro Shidoji[2]*

1. Department of Nutrition, Kiryu University, Gunma 379-2392, Japan; tabata-yu@kiryu-u.ac.jp (Y.T.), ORCID: 0000-0002-1117-5215
2. Molecular and Cellular Biology, Graduate School of Human Health Science, University of Nagasaki, Nagasaki 851-2195, Japan; shidoji@sun.ac.jp (Y.S.), ORCID: 0000-0002-2136-6752.
*Correspondence: shidoji@sun.ac.jp

Supplementary Table S1. The nucleotide sequences of each primer used for real-time RT-qPCR.

Genes	Primer	Sequence (5' – 3')
CYP1A1	F	CATCCCCCACAGCACAACA
	R	CAGGGGTGAGAAACCGTTCA
CYP1A2	F	AGTCCAGGAGCACTATCAGG
	R	CCTGCTCCAAAGATGTCATT
CYP1B1	F	GCCACTATCACTGACATCTTCGG
	R	CACGACCTGATCCAATTCTGCC
CYP2A6	F	CTCATGAAGATCAGTGAGCGCTAT
	R	CTCCCCGTTGCTGAATACCA
CYP2B6	F	CCACCCTAACACCCATGACC
	R	CCCAGGTGTACCGTGAAGAC
CYP2D6	F	TTCCTGCCTTTCTCAGCAGG
	R	CCAGGAAAGCAAAGACACCA
CYP3A4	F	TATTTTGTCCTACCATAAGGGCTTT
	R	AGCACAGGCTGTTGACCATC
CYP3A5	F	GTCTCTCTGTTTCCAAAAGATACC
	R	TGAAGAAGTCCTTGCGTGTC
28S rRNA	F	TTAGTGACGCGCATGAATGG
	R	TGTGGTTTCGCTGGATAGTAGGT

F: forward primer, R: reverse primer
CYP, cytochrome P450.

Supplementary Table S2. The condition of thermal cycler for real-time RT-PCR.

	Temperature, Duration	Slope
Denature	95ºC, 600 s	20ºC / s
PCR (40 cycles)	95ºC, 15 s	20ºC / s
	60ºC, 60 s	20ºC / s
Melting	95ºC, 0 s	20ºC / s
	57ºC, 15 s	20ºC / s
	98ºC, 0 s	-
Cooling	40ºC, 30 s	20ºC / s

Supplementary Table S3. The condition of thermal cycler for real-time RT-PCR of *28S rRNA*.

	Temperature, Duration	Slope
Denature	95ºC, 600 s	4.4ºC / s
PCR (40 cycles)	95ºC, 10 s	4.4ºC / s
	60ºC, 10 s	2.2ºC / s
	72ºC, 3 s	4.4ºC / s
Melting	95ºC, 1 s	4.4ºC / s
	65ºC, 15 s	2.2ºC / s
	95ºC, 1 s	-
Cooling	40ºC, 30 s	4.4ºC / s

Supplementary Table S4. The sequences of each siRNA used for knockdown experiments.

		Sequence (5' – 3')
siCYP3A4	Sense A	CCAGAGACCUCAAAUUACUtt
	Antisense A	AGUAAUUUGAGGUCUCUGGtt
	Sense B	CCAAUGGACUGCAUAAAUAtt
	Antisense B	UAUUUAUGCAGUCCAUUGGtt
	Sense C	CCAACUGUCUCGAUGCAAUtt
	Antisense C	AUUGCAUCGAGACAGUUGGtt

CYP, cytochrome P450.

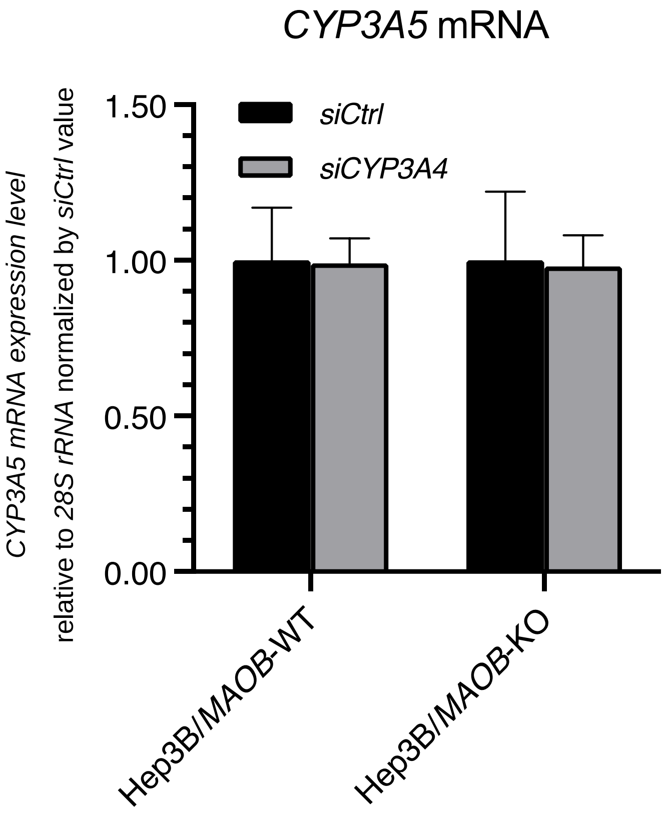

Supplementary Figure S1.

The expression levels of *CYP3A5* mRNA in Hep3B/*MAOB*-WT cells and Hep3B/*MAOB*-KO cells treated with *CYP3A4* siRNA for 72 h are shown as relative expression level to *CYP3A5* mRNA in *siCtrl*-treated Hep3B/*MAOB*-WT cells.

Each bar represents the mean ± SEM (n = 3).

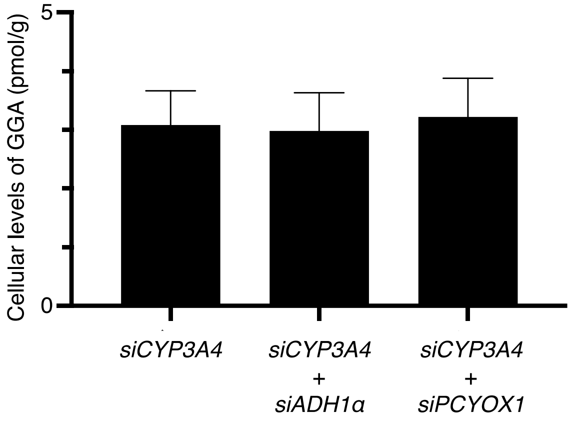

Supplementary Figure S2.

Cellular GGA levels in Hep3B/*MAOB*-KO cells treated with *CYP3A4* siRNA and *ADH1α* siRNA or *PCYOX1* siRNA. Each bar represents the mean ± SEM (n = 3).

(A)

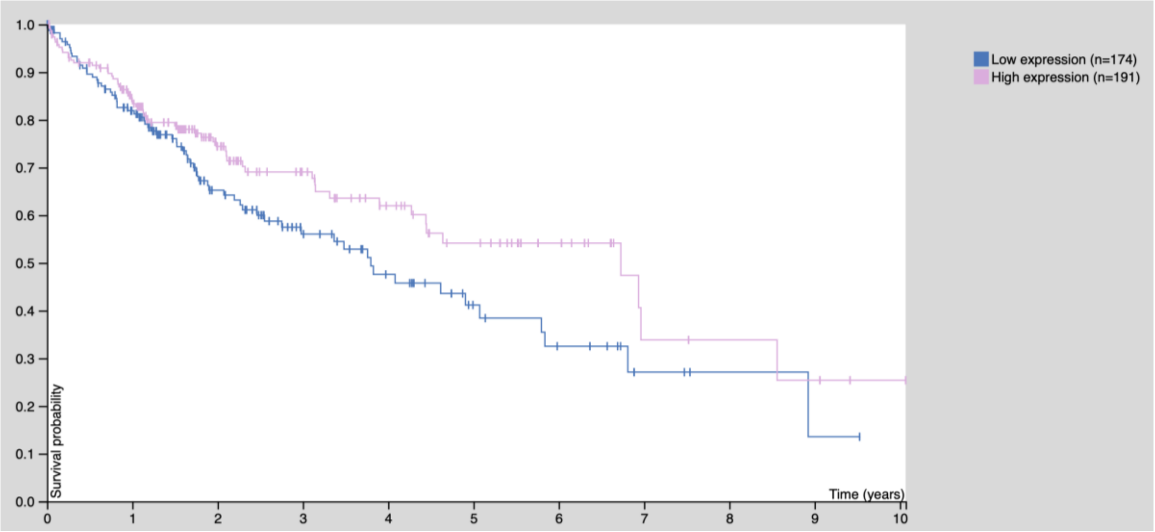

(B)

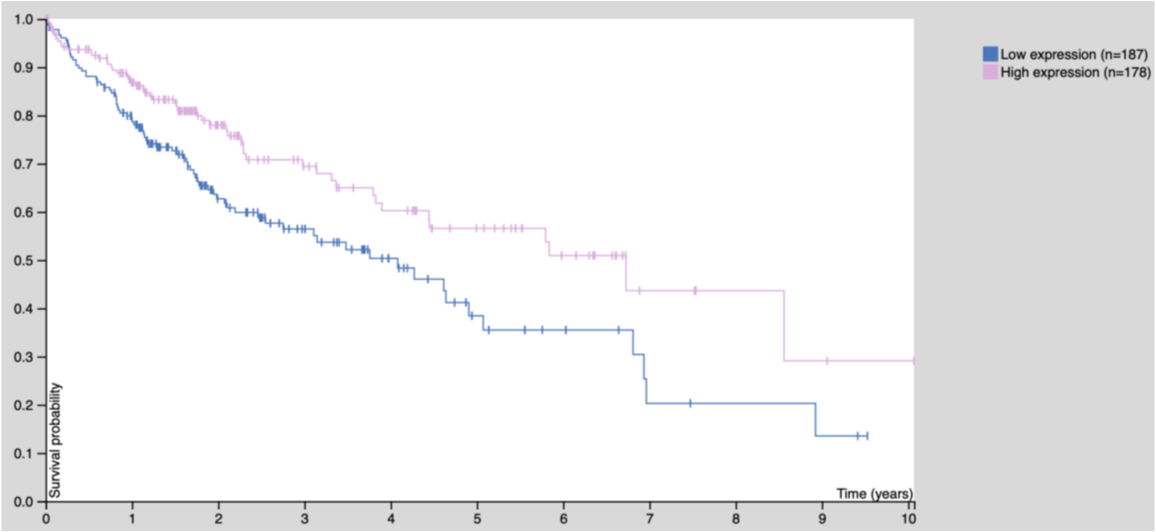

Supplementary Figure S3.

Prognosis of hepatoma patients between the high and low expression groups of the *MAOB* or *CYP3A4* gene shown by Kaplan-Meier plots.

Image credit: Human Protein Atlas, www.proteinatlas.org, ([32] Uhlén M et al, 2015).

Image available at the following URL:

https://v21.proteinatlas.org/ENSG00000069535-MAOB/pathology/liver+cancer (A) and

https://v21.proteinatlas.org/ENSG00000160868-CYP3A4/pathology/liver+cancer (B).
